# Case Report: The immune architecture of immunotherapy-induced cutaneous sarcoidosis resembles peritumoral inflammation

**DOI:** 10.3389/fimmu.2025.1432927

**Published:** 2025-03-03

**Authors:** Catherine J. Wang, Jennifer Strong, Margaret E. Gatti-Mays, Wiem Lassoued, Sam Sater, Julius Strauss, Jason M. Redman, Jeffrey Schlom, James L. Gulley, Isaac Brownell

**Affiliations:** ^1^ National Institute of Arthritis and Musculoskeletal and Skin Diseases, National Institutes of Health, Bethesda, MD, United States; ^2^ Laboratory of Tumor Immunology and Biology, National Cancer Institute, National Institutes of Health, Bethesda, MD, United States; ^3^ National Cancer Institute, National Institutes of Health, Bethesda, MD, United States

**Keywords:** immunotherapy, anti-PD-L1, avelumab, cutaneous sarcoidosis, immune-related adverse event, case report

## Abstract

Avelumab, is an anti-PD-L1 immune checkpoint inhibitor (ICI). Like other ICI, avelumab can cause immune-related adverse events. Although rare, sarcoidosis-like granulomatous reactions have been described in patients on anti-CTLA-4 and anti-PD-1 immunotherapy. Here we report a case of treatment emergent cutaneous sarcoidosis in a patient who received avelumab for metastatic colon cancer. A 56-year-old African American woman with metastatic colon cancer that had progressed after multiple lines of treatment, including other immunotherapy agents, was enrolled on a clinical trial with avelumab. While on treatment, the patient developed two skin lesions, and histopathological examination of both biopsies demonstrated chronic granulomatous inflammation in the dermis with multinucleated giant cells containing asteroid bodies, consistent with cutaneous sarcoidosis. Multiplex immunofluorescence revealed parallels between the immune architecture of the patient’s cutaneous sarcoidal lesion and an excised tumor metastasis. Recognizing cutaneous sarcoidosis as a rare adverse effect of ICI immunotherapy is important because sarcoidal lesions can be mistaken for metastatic disease on clinical exam and medical imaging. We noticed similar immune composition of the sarcoidal granuloma and tumor microenvironment. However, further studies are needed to fully elucidate the mechanism of ICI associated sarcoidosis.

## Introduction

Immune checkpoint inhibitors (ICIs) have revolutionized cancer therapy (1). Blocking these checkpoints promotes host immune responses against tumors by inhibiting suppressive signals to T-lymphocytes (2).

By blocking immune inhibition, ICIs can induce autoimmunity and cause immune-related adverse events (irAEs). While the mechanisms of irAE are not fully understood, checkpoint inhibitors drive a variety of inflammatory responses, including activation of the T-helper cells 17 (Th17) pathway and autoreactive T cells (3, 4). Some of the more commonly affected organs are the colon, liver, skin, thyroid, and pituitary (5). Immune-related toxicities may also be associated with durable therapeutic responses in some cases (6, 7). Recently, sarcoidosis or sarcoidosis-like granulomatous reactions have been described as rare irAEs in patients on ICIs. There are several cases of patients on the anti-CTLA-4 antibody ipilimumab who developed biopsy-confirmed sarcoidosis-like reactions in the lungs, skin, lymph nodes (8–10), and one case in the brain (11). Anti-PD-1 agents such as nivolumab and pembrolizumab have also been implicated in the development of sarcoidosis-like adverse events (12–14). Avelumab is an anti-PD-L1 antibody approved for the treatment of metastatic Merkel cell carcinoma, urothelial carcinoma, and, in combination with axitinib, renal cell carcinoma (15). There are few cases reporting sarcoidosis in patients treated with PD-L1 inhibitors (16–19) – however, to our knowledge, there are no cases in the literature describing isolated cutaneous sarcoidosis lesions in patients on anti-PD-L1 therapy. Herein, we report a case of cutaneous sarcoidosis in a patient with colon cancer receiving the anti-PD-L1 antibody avelumab and use multiplex immunofluorescence (MxIF) to compare the immune architecture of the patient’s sarcoidal lesion with that of their tumor.

## Case summary

**Figure 1:**
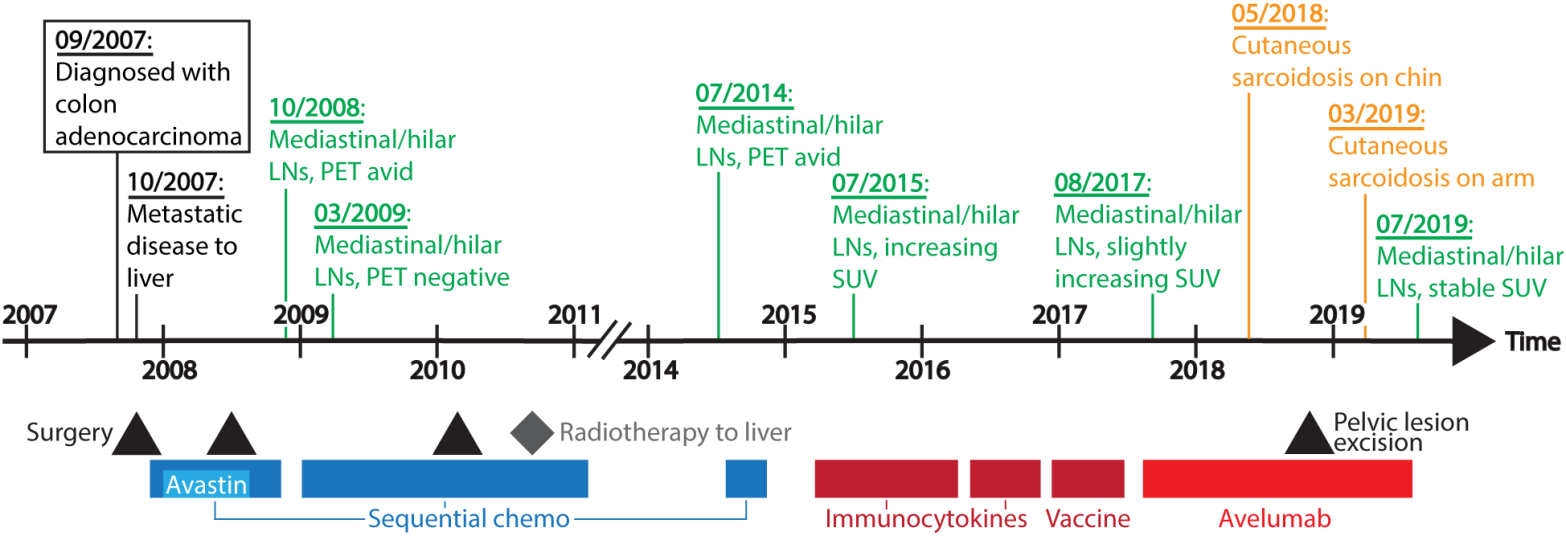
Timeline of disease course and treatment. Systemic treatment interventions are represented in colored bars, with sequential chemo (chemotherapy including FOLFOX, FOLFIRI, capecitabine, and irinotecan) in dark blue, bevacizumab (Avastin) in light blue, other immunotherapy agents (immunocytokines and vaccine) in dark red, and avelumab in red. Black triangles indicate when the patient underwent surgery, and a grey diamond depicts when the patient received radiation therapy. Above the timeline are dates of significant clinical events. LNs, lymph nodes; PET, positron emission tomography; SUV, standardized uptake values.

A 56-year-old African American woman with KRAS mutated, microsatellite stable colon adenocarcinoma metastatic to the liver, retroperitoneum, and hilar and mediastinal lymph nodes was enrolled on a phase I clinical trial (JAVELIN Solid Tumor, NCT01772004) evaluating the safety of the anti-PD-L1 antibody avelumab in August 2017 (**Figure 1**). Per protocol, the patient received weekly avelumab monotherapy at a dose of 10mg/kg for 12 doses followed by treatment every 2 weeks until disease progression. The drug was tolerated well with only a mild infusion reaction during the first treatment. Restaging scans after seven weekly doses demonstrated mildly enlarging mediastinal and hilar adenopathy, while her retroperitoneal lymphadenopathy remained stable. Per Response Evaluation Criteria in Solid Tumors (RECIST) version 1.1, she had stable disease and therefore continued on treatment. Subsequent scans confirmed stable disease.

**Figure 2:**
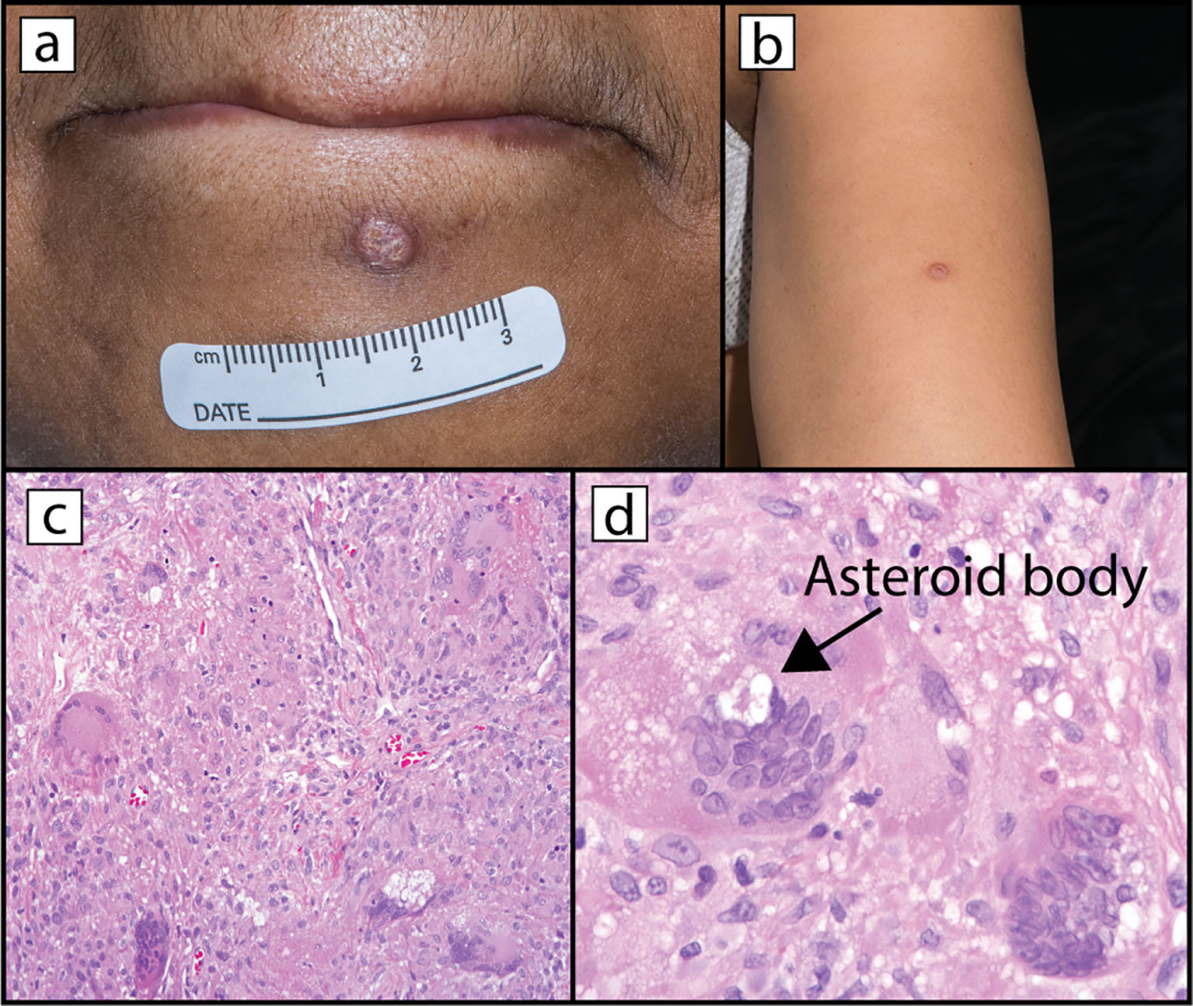
Clinical presentation and histopathology of cutaneous sarcoidosis lesions. The patient presented after 30 treatments of avelumab with a 7-mm keratotic papule on her left mid chin **(a)**. The lesion was removed via shave biopsy but recurred several months later. Eight months later, the patient developed a second papule **(b)** on her left upper arm that was removed via punch biopsy. Hematoxylin and eosin staining revealed well-formed granulomas that included multinucleated giant cells [**(c)**– 100X original magnification]. Many giant cells contained asteroid bodies [**(d)** – 400X original magnification]. Stains for microorganisms were all negative (GMS, AFB, Fite, PAS-D, AFB, Ziehl-Neelsen).

In July 2018, after 30 doses of avelumab, the patient developed a 7-mm papule with surrounding hyperpigmentation on her left chin (**Figure 2a**), which she stated appeared approximately two months prior. She reported that it had grown in size and endorsed manipulating the lesion. She experienced some pruritus at the site but denied discharge, drainage, or other additional dermatological complaints. A shave biopsy was performed, and pathology demonstrated non-necrotizing chronic granulomatous inflammation with multinucleated giant cells consistent with a sarcoidosis-like reaction. Shortly thereafter, treatment with the study drug was suspended due to palliative surgical resection of a pelvic mass. Pathology of the pelvic mass was consistent with metastatic adenocarcinoma, and there was no evidence of granulomas. After surgical recovery, the patient was restarted on avelumab. After five more doses of avelumab, the patient returned to clinic with a new lesion on her left upper arm (**Figure 2b**). On exam, there was a 6-mm, round, crusted, translucent papule with white scar-like features and prominent blood vessels on dermoscopy. She also had interval regrowth of the previously biopsied papule on her left chin. Punch biopsy of the lesion on the left arm demonstrated well-formed granulomas without necrosis and containing multinucleated giant cells with asteroid bodies (**Figures 2c, d**). The tissue stained negative for fungal, bacterial, or acid-fast organisms. Polarized microscopy was negative for foreign bodies.

Due to concerns for systemic sarcoidosis in light of these cutaneous lesions and in the setting of her known mediastinal lymphadenopathy, blood angiotensin-converting enzyme (ACE) levels were evaluated and found to be within normal limits (47.9 U/L, normal range 3 to 52 U/L). Calcium levels were consistently within the normal range. Throughout the trial, the patient denied any respiratory symptoms and had no personal or family history of sarcoidosis.

Prior to this clinical trial, the patient had been enrolled on three other trials with immunotherapy agents – two trials with immunocytokines and one trial with a cancer vaccine. The patient tolerated all previous immunotherapy agents without significant toxicity or side effects, and the reason for treatment cessation in all cases was due to disease progression.

**Figure 3:**
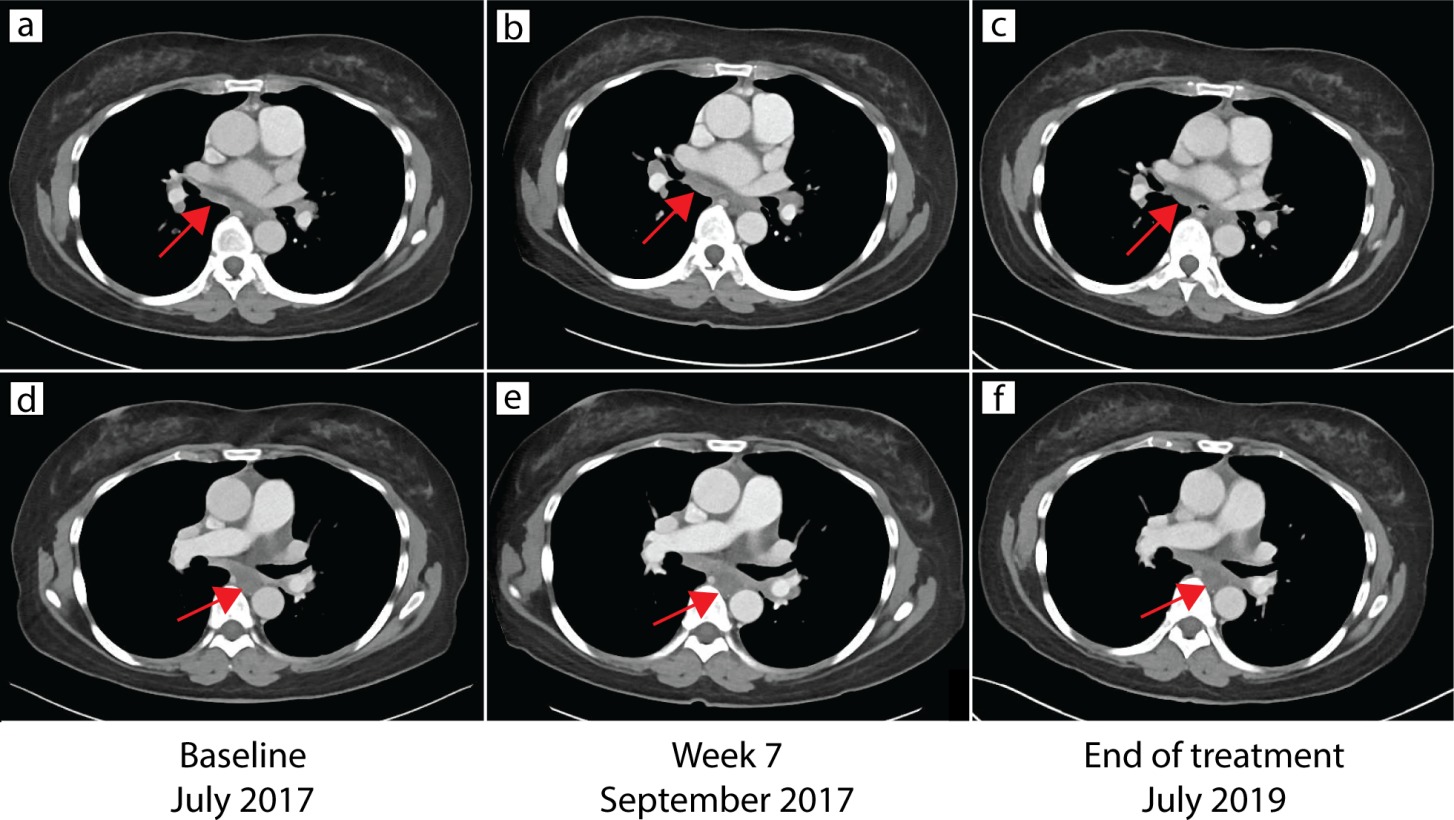
CT of chest demonstrating stable mediastinal lymphadenopathy. CT of the chest at baseline in July 2017 **(a, d)**, week 7 in September 2017 **(b, e)**, and end of treatment in July 2019 **(c, f)** demonstrated stable mediastinal lymphadenopathy (arrows).

The patient’s mediastinal lymphadenopathy remained unchanged after the initial increase in September 2017 (**Figure 3**), suggesting these were metastatic carcinoma lesions and not pulmonary sarcoidosis. Due to the timing of these skin lesions and their characteristic histopathology, the patient was given the diagnosis of immunotherapy-induced cutaneous sarcoidosis. As the patient expressed distress at the appearance of her persistent chin lesion, she was prescribed topical fluocinonide 0.05% ointment once daily. At last follow up four months later, the chin and arm lesions remained stable. Her metastatic colon cancer remained stable for the duration of the two-year (56 dose) treatment protocol on avelumab.

## Immunophenotyping

**Figure 4:**
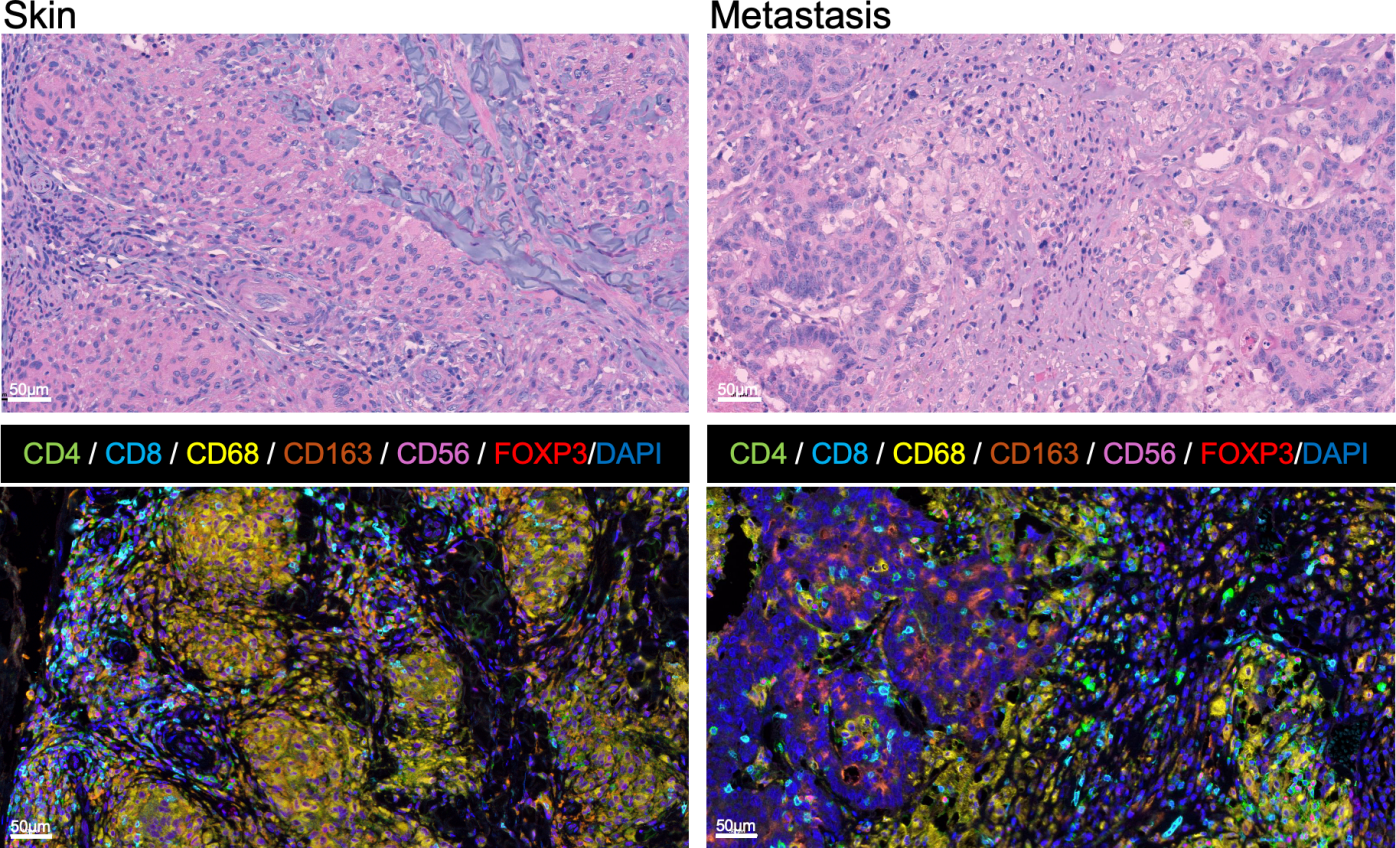
Cytological and immune architecture of immune-related sarcoidal granuloma and resected colon cancer metastasis. Histological sections with hematoxylin and eosin and multiplex immunofluorescence (MxIF) staining from the patient’s skin lesion (left) and tumor metastasis (right). In contrast to the dense sarcoidal granulomas surrounded by lymphocytic infiltrates in the skin, the tumor is associated with a more mixed inflammatory infiltrate containing looser nests of macrophages. MxIF Opal method with multiple antibodies (CD4, CD8, CD68, CD163, CD56, FOXP3) and counterstained with DAPI identified the presence of CD68+ histiocytes/macrophages (yellow) including a subset of immunosuppressive CD163+ M2 macrophages (orange). The remaining immune cells (ICs) included inflammatory T-helper [CD4+, FOXP3- (green)], natural killer (NK) [CD56+ (purple)], and cytotoxic T lymphocytes (CTL) [CD8+ (blue)], as well as immunosuppressive Tregs [CD4+, FOXP3+ (red nuclei)].

Compared to the sarcoidal granulomas in the skin, histological examination of the tumor metastasis revealed a more mixed immune infiltrate containing disorganized nests of histocytes (**Figure 4**). To study the immune architecture of the patient’s sarcoidal lesion, we performed MxIF assays on formalin fixed paraffin embedded (FFPE) sections of the patient’s skin lesion and resected pelvic metastasis (**Figure 4**, **Supplementary Methods**). Consistent with the hematoxylin and eosin histopathology, the patient’s cutaneous lesion consisted primarily of granulomas with CD68+ histiocytes/macrophages (**Figure 4**, **Supplementary Figure S1**). MxIF demonstrated the presence of CD4+ T cells, cytotoxic CD8+ T cells, CD56+ natural killer (NK) cells, and regulatory T-cells (Tregs) at the periphery of some granulomas. Although the patient’s pelvic metastasis did not contain sarcoidal granulomas, we noticed that tumor associated macrophages formed nests around the tumor that were surrounded and infiltrated by other immune cells (ICs). Unlike the sarcoidal granulomas, tumor associated macrophages were less epithelioid and did not form giant cells.

Quantification of MxIF stained cells revealed that, compared to the metastatic tumor lesions, the sarcoidal granulomas contained higher densities of CD68+ macrophages and had significantly more M2 macrophages in both the lesional granulomas and perilesional stroma (**Supplementary Figure S2**). The granulomas also contained significantly more CD8+ T cells compared to the metastasis. When the perilesional regions were compared, the stroma of the sarcoidal lesion contained significantly more NK cells than that of the pelvic metastasis. The full implications of these immune architecture patterns during anti-PD-L1 therapy are unclear and require further research.

## Discussion and conclusions

Sarcoidosis is a chronic, multisystem disease of unknown etiology that is histologically characterized by non-necrotizing epithelioid-cell granulomas (20). It commonly involves the lung, skin, and eye and can also affect the liver, spleen, central nervous system, heart, and bone (20). Sarcoidosis is suspected in patients with distinctive clinical (e.g. fatigue, weight loss, shortness of breath, skin changes, vision changes) and radiographic features (e.g. hilar and mediastinal lymphadenopathy) and is typically confirmed with histopathologic assessment of a tissue biopsy. Lab abnormalities, such as elevated calcium or ACE, may also be seen in this disease.

There have been several reports of the development of sarcoidosis-like lesions during anti-CTLA-4 and anti-PD-1 therapy. Mediastinal and hilar lymphadenopathy are the most commonly reported manifestations of immunotherapy-induced sarcoidosis (14, 21). The patient’s scans showed multiple pulmonary, hilar and mediastinal lesions that were interpreted as part of her cancer disease burden. However, as these nodes have never been biopsied, it is possible that any of them could represent immunotherapy-induced sarcoidosis or baseline idiopathic sarcoidosis.

After the patient’s diagnosis of cutaneous sarcoidosis, previous scans were re-evaluated to determine if she had occult sarcoidosis. In 2009, while on chemotherapy, the patient had resolution of mediastinal/hilar lymph node metabolic activity. At the time of clinical progression of her metastatic colon cancer in July 2014, a PET/CT demonstrated increased avidity in mediastinal/hilar lymph nodes. Both sarcoidosis and metastatic pulmonary lesions can show increased uptake of 18F-FDG on PET/CT (22). Between 2015 to 2016, hilar and mediastinal lymph nodes had increasing standardized uptake values (SUV) up to 26, while pulmonary lesions had maximum SUV values to 16 and abdominal/retroperitoneal lymph nodes ranged from 3 to 11. Shortly after avelumab initiation in August 2017, her CT scan demonstrated a small increase in hilar/mediastinal lymphadenopathy that then remained stable throughout treatment. Her abdominal and retroperitoneal lymphadenopathy generally remained unchanged throughout avelumab treatment. As the patient’s hilar/mediastinal lymphadenopathy were present before ICI initiation and followed the same clinical trajectory as her other metastatic disease, we think it is unlikely that these nodes represent asymptomatic sarcoidosis. However, without a biopsy, a diagnosis cannot be confirmed.

In the reported literature of anti-CTLA-4 or anti-PD-1-induced sarcoidosis, patients can present with cutaneous sarcoidosis either with involvement of other sites, or less commonly as the only affected site (12, 14, 21). This patient confirms that PD-L1 inhibition is also capable of inducing sarcoidosis-like granulomatous reactions that can present as cutaneous disease. Furthermore, this case supports that these sarcoidal reactions can have delayed presentations. In previously reported cases, sarcoidosis-like granulomatous reactions presented up to 6 months after anti-PD-L1 therapy initiation (19). Interestingly, ICI-related granulomatous-like reactions may be associated with a favorable response to cancer therapy (23). However, in a separate review of patients who developed sarcoid-like reactions while on ICI therapy, comparable numbers of patients achieved favorable outcomes to those who developed disease progression. (24) Given these conflicting findings, it is unclear how the diagnosis of ICI-related sarcoidosis might impact the prognosis of this case. It is important to recognize sarcoidosis as a possible irAE in ICI treatment as sarcoidal lesions can mimic disease progression or metastases. Mistaking sarcoidosis for disease progression may falsely prompt physicians to prematurely halt or alter potentially life-saving cancer treatment.

Treatment for sarcoidosis-like granulomatous reactions varies. For idiopathic sarcoidosis, patients may not be limited by their disease, and the decision to treat is based on the risks and benefits of starting therapy. Treatment options include corticosteroids, immunosuppressive drugs such as methotrexate or hydroxychloroquine, or anti-tumor necrosis factor alpha (TNF-α) agents (20). Strategies for treating immunotherapy-induced sarcoidosis include topical or systemic corticosteroids, hydroxychloroquine, switching immunotherapy agents, or halting immunotherapy treatment altogether (12, 14, 25). Balestra and colleagues described a case of pulmonary sarcoidosis following initiation of avelumab therapy for melanoma (16). Treatment for the patient’s pulmonary sarcoidal lesions was not required as the patient was asymptomatic from these lesions. In another case, Mitchell and associates described a patient with metastatic urothelial cancer who developed intrathoracic sarcoidosis as an adverse effect to atezolizumab, another anti-PD-L1 drug (17). Due to the progression of the patient’s lymphadenopathy, atezolimumab was held and the patient was started on prednisone. In our case, the patient was asymptomatic from her cutaneous sarcoidosis but disliked the appearance of the lesion on her face and was prescribed a topical corticosteroid ointment. The decision to begin therapy for sarcoidosis-like granulomatous reactions and/or hold immunotherapy treatment should be a shared decision between physician and patient based on the severity of the sarcoidosis-like disease, the likelihood of cancer benefit, and degree of patient distress.

The exact pathophysiology of sarcoidosis-like reactions appearing after immunotherapy treatment is still under investigation. It has been hypothesized that CTLA-4 may play an important role in sarcoidosis-like adverse event development. One study found decreased levels of CTLA-4 expression on activated Tregs and Th17 cells in mediastinal lymph nodes of sarcoidosis patients compared to healthy controls (26). Th17 cells were also increased in proportion to Treg cells. This may contribute to Th17 activation and impaired Treg-mediated immune suppression, leading to an abnormal autoimmune response – an important factor in sarcoidosis pathology. Our MxIF analysis did not quantify how many CD4+ lymphocytes were Th17 cells in the patient, but overall CD4+ cells were proportionally more abundant than Tregs. Another study investigating the role of PD-1/PD-L1 in sarcoidosis pathogenesis (27) found that sarcoidosis patients had elevated levels of PD-1+ CD4+ T cells systemically and upregulated levels of PD-L1 on granuloma cells. Inhibiting the PD-1 pathway enhanced the proliferative capacity of the CD4+ T cells in sarcoidosis patients to similar levels as healthy controls, suggesting that PD-1 may play a significant role in sarcoidosis pathogenesis. Furthermore, blockade of the PD-1/PD-L1 pathway has been shown to activate mTOR signaling in macrophages, which could induce granuloma formation. (24) The precise relationship between PD-1/PD-L1 and the development of immunotherapy-induced sarcoidosis requires further investigation.

In conclusion, patients on PD-L1 blockade may present with the rare irAE of cutaneous sarcoidosis. In our experience, local topical treatment in otherwise asymptomatic patients is sufficient to treat these benign cutaneous lesions without the need for treatment cessation of potentially curative ICI therapy. MxIF of the cutaneous sarcoidosis lesions compared to malignant tissue from the same patient offered insights into the immune responses underlying these distinct entities. Cutaneous sarcoidosis is an autoimmune granulomatous process which was characterized by tight clusters of M2 predominant macrophages with surrounding inflammatory infiltrate. The tumor metastasis, in contrast, largely had a mixed inflammatory infiltrate with fewer M2 macrophages forming loose nests. Despite these differences, the similarities in the spatial distribution and lymphoid composition of immune cells in the periphery of the cutaneous sarcoidal granulomas and the tumor microenvironment are intriguing and support further investigations into the parallels between tumor immune responses and sarcoidal adverse events.

## Data Availability

The original contributions presented in the study are included in the article/**Supplementary Material**. Further inquiries can be directed to the corresponding author/s.
