# Supplementary material for: Case Report: The immune architecture of immunotherapy-induced cutaneous sarcoidosis resembles peritumoral inflammation
Source: Front Immunol. 2025 Mar 3;16:1432927. doi: 10.3389/fimmu.2025.1432927 (PMC11914793; doi:10.3389/fimmu.2025.1432927)
Supplement: Supplementary file 1 [file DataSheet1.docx]

Supplementary Material

**Supplementary Methods**

Formalin fixed paraffin embedded (FFPE) slides of the patient’s cutaneous sarcoidal lesion and tumor metastasis were stained using multiplex immunofluorescence (MxIF) and TSA-Opal technology. Opal multiplexing is a serial immunofluorescence method that relies on tyramide signal amplification, which creates an amplification of signal that then covalently binds to the epitope in a specific manner. Primary and secondary antibody complexes are subsequently removed for serial immunofluorescence, while the covalent fluorescent signal remains. Single controls and an unstained slide were stained with each group of slides. Antibodies applied to the tissue samples in this study are shown in the table below. Slides from H&E stain and MxIF were scanned at 20X magnification, using PhenoImager (Akoya Biosciences). Representative regions of interest (ROI) were each 0.16 mm^2^ and scanned at 40X magnification for image analysis. Twelve ROI were selected from the skin tissue while eleven were selected from viable metastatic tissue. Markers and phenotypes were quantified using Inform software (Akoya Biosciences). ROI were parsed into lesional and stromal areas by a trained machine learning algorithm. Cell types were identified by the following markers: CD4 T cells (CD4+/CD68-/CD163-/FOXP3-), CD8 T cells (CD8+), regulatory T cells (Treg) (CD4+/CD68-/CD163-/FOXP3+), natural killer (NK) cells (CD56+), macrophages (CD68+) and M2 macrophages (CD68+/CD163+). The percentage of M2 macrophages was calculated by taking the ratio of M2 cells to total macrophages and is reported as a percentage. Statistical comparisons of cell densities and percentages in the perilesional and lesional regions of the skin and tumor metastasis were performed using a one-tail, two-sample T-test.

| **Antibody** | **Vendor** | **Catalog #** | **Dilution** | **Host** | **HIER** | **Secondary** | **Opal** |
| --- | --- | --- | --- | --- | --- | --- | --- |
| CD4 | Abcam | ab133616 | 1:1000 | Rabbit | ER1 | MACH2 (biocare medical, cat no:RHRP520) | 520 |
| CD68 | Cell Signaling | D4B9C | 1:400 | Rabbit | ER1 | Akoya Opal Polymer (Akoya Biosciences cat no. ARH1001EA) | 570 |
| CD8 | Abcam | ab215041 | 1:1000 | Rabbit | ER2 | Akoya Opal Polymer (Akoya Biosciences cat no. ARH1001EA) | 480 |
| CD163 | Abcam | ab182422 | 1:200 | Rabbit | ER2 | Akoya Opal Polymer (Akoya Biosciences cat no. ARH1001EA) | 620 |
| CD56 | Sigma-Aldrich | 156R-97 | 1:250 | Rabbit | ER1 | Akoya Opal Polymer (Akoya Biosciences cat no. ARH1001EA) | 690 |
| FOXP3 | Invitrogen | MA5-16365 | 1:200 | Rabbit | ER2 | MACH2 (biocare medical, cat no:RHRP520) | 780 |

**Supplementary Table 1**. **Multiplex immunofluorescence antibody panels.** Antibodies applied to tissue samples of the patient’s cutaneous sarcoidal lesion and tumor metastasis. HIER, heat-induced epitope retrieval.

**
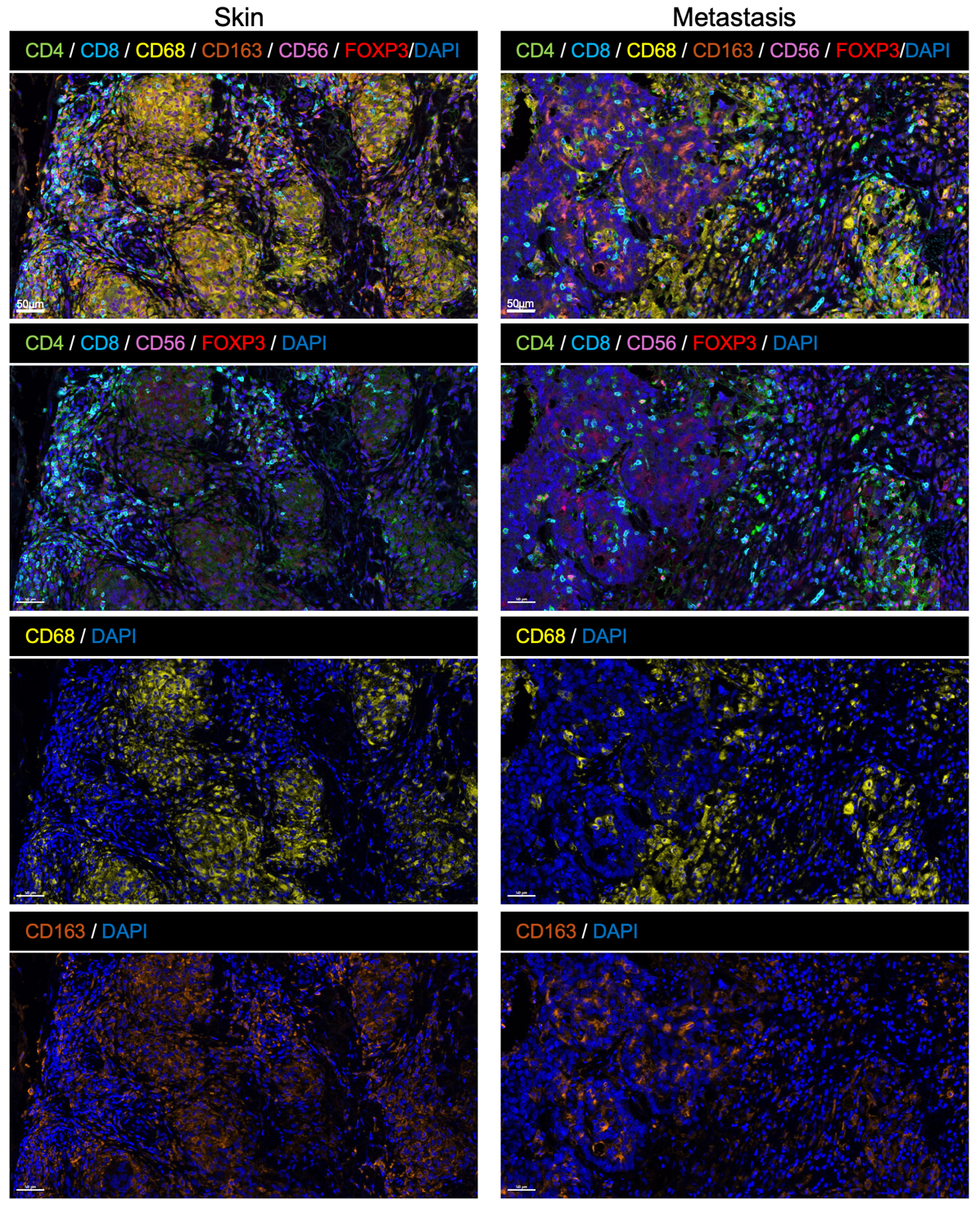
**

**Supplementary Figure 1. Immune architecture of immune-related sarcoidal granuloma and resected colon cancer metastasis from Figure 4.** Multiplex immunofluorescence (MxIF) images from the patient’s skin lesion (left) and tumor metastasis (right) are shown. MxIF Opal method with multiple antibodies (CD4, CD8, CD68, CD163, CD56, FOXP3) and counterstained with DAPI. Staining for CD4, CD8, CD56, and FOXP3 highlights the lymphocytic and NK cell infiltrates seen in the periphery of the skin granulomas and interspersed throughout the tumor metastasis. Macrophages [CD68+ (yellow)] and M2 macrophages [CD163+ (orange)] are seen within the sarcoidal granuloma and in association with the tumor metastasis.


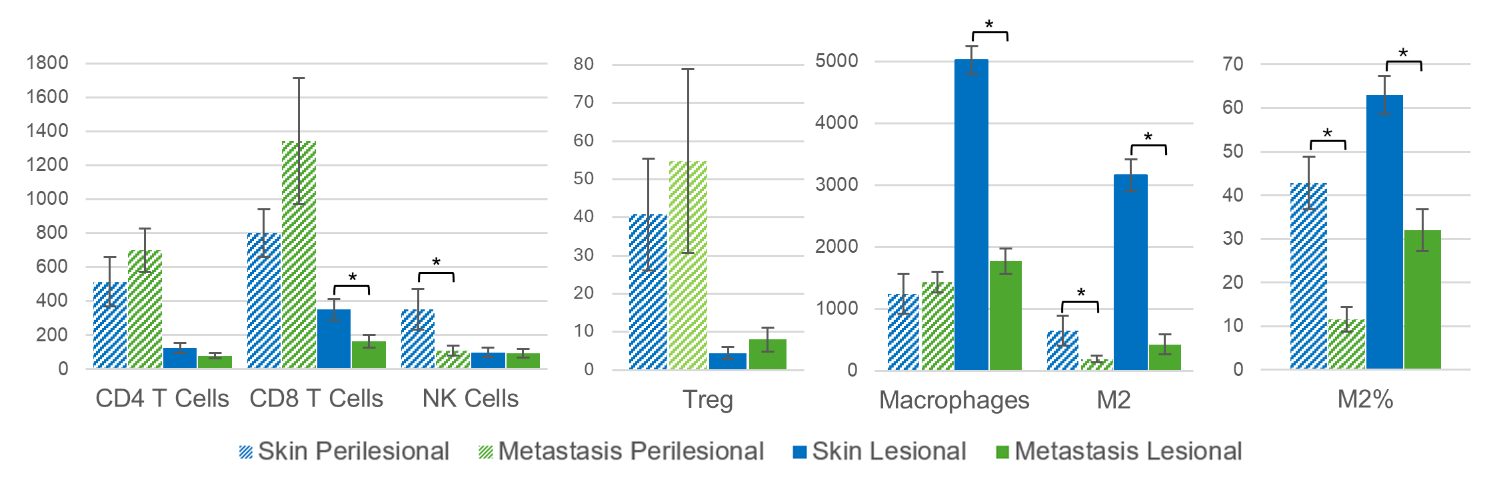


**Supplementary Figure 2**. **Multiplex immunofluorescence (MxIF) stained cell densities (cells/mm^2^) of the immune-related sarcoidal granuloma and resected colon cancer metastasis.** Perilesional skin contained significantly higher densities of natural killer (NK) cells and M2 macrophages compared to the perilesional region of the tumor metastasis. The sarcoidal granulomas contained significantly higher densities of CD8 T cells, macrophages, and M2 macrophages compared to the tumor metastasis. NK, natural killer; Treg, regulatory T cells; *, p<0.05.
